# Association of response to hepatitis B vaccination and survival in dialysis patients

**DOI:** 10.1186/1471-2369-13-97

**Published:** 2012-08-30

**Authors:** Shih-Yi Lin, Jiung-Hsiun Liu, Shu-Ming Wang, I-Kuan Wang, Chen-An Tsai, Yao-lung Liu, Hsin-Hung Lin, Chiz-Chung Chang, Chiu-Ching Huang

**Affiliations:** 1 Graduate Institute of Clinical Medical Science, College of Medicine, China Medical University, Taichung, Taiwan; 2 Division of Nephrology and Kidney institute, China Medical University Hospital, Taichung, Taiwan; 3 School of Medicine, China Medical University, Taichung, Taiwan; 4 Department of Agronomy, National Taiwan University, Taipei, Taiwan

**Keywords:** Hepatitis B vaccination, Immune response, Post-vaccination anti-HBs titers

## Abstract

**Background:**

The status of immunocompromised patients is well recognized in end stage renal disease (ESRD). As described recently, this acquired immune dysfunction in the uremic milieu may be one of the main pathogenic factors for mortality in ESRD. The aim of this study was to determine the relationship between the immune response following a hepatitis B vaccination (HBV vaccination) and the survival of maintenance dialysis patients.

**Methods:**

A total of 156 patients (103 on hemodialysis and 53 on continuous ambulatory peritoneal dialysis) were recruited. After receiving a full dose of the HBV vaccination, all patients were followed up for to 5 years to evaluate the association of patient survival, cause of mortality, and immune response.

**Results:**

The response rate to the hepatitis B vaccination was 70.5%. There was no significant association between the immune response and the 5-year survival rate (p =0.600) or between the post-vaccination anti-HBs titers and the 5-year survival rate (p = 0.201). The logistic prediction model with the coefficient as non-response following HBV vaccination, diabetes mellitus, old age, and low albumin level could significantly predict infection-cause mortality (sensitivity = 0.842, specificity = 0.937).

**Conclusion:**

There was no significant association between the immune response to HBV vaccination and the 5-year survival rate. However, non-response following HBV vaccination might be associated with infection-cause mortality in dialysis patients.

## Background

End-stage renal disease (ESRD) significantly increases the risk of mortality and morbidity. The leading cause of death in ESRD is cardiovascular disease (CVD), which accounts for more than half of deaths in uremic patients, with infection the second leading cause [1,2]. Previous studies have investigated the relationships among endothelial dysfunction, inflammation, and atherosclerotic vascular disease in chronic kidney disease [3,4]. Uremic-related inflammation, which commonly occurs during pre-dialysis and persists even after dialysis, is now regarded as an important predictor of cardiovascular and all-cause mortality in the ESRD population [5]. Currently, it is speculated that acquired immune dysfunction in the uremic milieu may be one of the main pathogenic factors, mediated through CVD and infections, in most ESRD-related deaths. Concomitant immunosuppression and immune activation in uremia would predispose patients to infection and CVD, respectively [6].

Although it is well established that aging, diabetes mellitus (DM), and the presence of co morbid conditions predict a poor prognosis in ESRD, few studies have investigated immune dysregulation as a determinant of mortality in ESRD [7]. Furthermore, acquired immune dysfunction in uremic patients has various clinical features, ranging from proness to infection to impaired immune response to vaccinations. The aim of our study was to link the clinical immune parameter, immune response and anti-HBs titers one month following HBV vaccination with mortality in ESRD populations.

## Methods

This clinical study complied with the Declaration of Helsinki and was approved by the Medical Ethics Committee of the China Medical University Hospital, Taichung (Taiwan, ROC).

This was a retrospective study of ESRD patients who underwent dialysis therapy at the dialysis unit of the China Medical University Hospital (Taichung, Taiwan) from March 2002 to March 2008 and who were followed up until March 2009.

The HBV vaccine was offered to all patients who tested negative for the HBV surface antigen (HBs Ag) and anti-HBs and who had not previously received the HBV vaccination. We excluded patients who had malignancies, who were receiving immunosuppressive agents, or who did not receive all four vaccinations. All patients were given four doses (40 μg per dose) of the hepatitis B vaccine (Engerix-B, GlaxoSmithKline Biologicals, Philadelphia, USA) in the deltoid muscles at 0, 1, 2, and 6 months. Anti-HBs titers were measured by an ELISA kit (AUSAB-EIA, Abbot Labs, Chicago, USA) one month after the final dose and annually thereafter.

We defined responders and non-responders according to the level of anti-HBs one month after the final injection (non-responders: < 10 IU/L; responders: ≥ 10 IU/L). These patients were followed up to assess the relationship between the initial immune response to vaccination and the 5-year-survival. In addition, over the 5-year follow-up period, we assessed the effect of one-month post-vaccination anti-HBs titers on survival.

To determine the factors related to HBs antibody formation following vaccination, we recorded the patient age, gender, presence of diabetes mellitus (DM), hemoglobin, serum albumin, triglyceride, cholesterol, fasting blood sugar, presence of hepatitis C antibodies, and dialysis modality at the initiation of vaccination [8-10].

### Statistical analysis

All statistical tests were performed with SPSS (version 15.0). A P value < 0.05 was considered statistically significant. For categorical variables, we used the Chi-squared test and presented data as absolute numbers or percentages. For continuous variables, we used Student’s *t*-test and presented the data as means ± standard deviations. The Cox regression model was used to investigate the association between seroconversion and mortality and the relationship between post-vaccination anti-HBs titers and mortality.

We adjusted for age, level of albumin, presence of DM, dialysis modality, and gender to determine the adjusted hazard ratios with the immune response to mortality.

We adapted the logistic regression model as follows: β0+ β1*Non-response + β2*(presence of DM)+ β3*Age+β4*Albumin+β5*(presence of DM)*Albumin + β6*Age*Albumin. We used this equation to evaluate the effects of non-response, diabetes mellitus, age, and the level of albumin in predicting mortality due to infection.

## Results

A total of 156 patients (64 males, 92 females) were recruited, with 110 responders and 46 non-responders (Table 1). Mean ages were higher for non-responders (62.63 ± 9.95 years) than for responders (55.75 ± 14.31 years, P = 0.003). The prevalence of diabetes or anti-HBV vaccination at the start of the study was 32.0%.

**Table 1:** Baseline characteristics of HBV vaccine responders and non-responders

**Characteristic**	**Non-responders**	**Responders**	**P value**
Number of pts.	46	110	
Age	62.63 ± 9.95	55.75 ± 14.31	0.003
Gender(M/F)	17/29	47/63	0.507
DM	19	31	0.111
HCV infection	9 (19.6%)	15 (13.6%)	0.353
Hemoglobin(g/dL)	9.49 ± 1.42	9.55 ± 1.38	0.786
Albumin(g/dL)	3.47 ± 0.37	3.50 ± 0.39	0.696
Dialysis modality (HD:PD)	31:15	72:38	0.817
Cholesterol(mg/dL)	184.71 ± 50.07	181.33 ± 42.66	0.669
Triglyceride(mg/dL)	187.93 ± 131.87	176.58 ± 124.46	0.610

Note: data are shown as the number (%) or mean ± SD, as appropriate.

Abbreviations: M, male; F, female.

Univariate analysis indicated no significant differences between responders and non-responders in terms of gender, presence of DM, presence of hepatitis C antibodies, hemoglobin, albumin, dialysis modality, triglyceride, cholesterol, and several other relevant factors (Table 1). By the time of the 5-year follow up, 35 patients had died. The most common cause of death was infection (54.3%; 19 patients, including 8 cases of severe pneumonia, 4 cases of peritonitis, 2 cases of infective endocarditis, and 1 case of either meningitis, diabetic foot infection, septic arthritis, urosepsis, and septicemia), followed by cardiovascular events (25.7%, 9 patients), gastrointestinal bleeding (5.7%, 2 patients), gastrointestinal perforation (5.7%, 2 patients), and others (8.6%, 3 patients). By the end of the study, the prevalence of DM in all available subjects (including those that died during the study) was 30%, but the impact of DM on the loss of anti-HBs Ab was not significant (p = 0.308).

**Figure 1:**
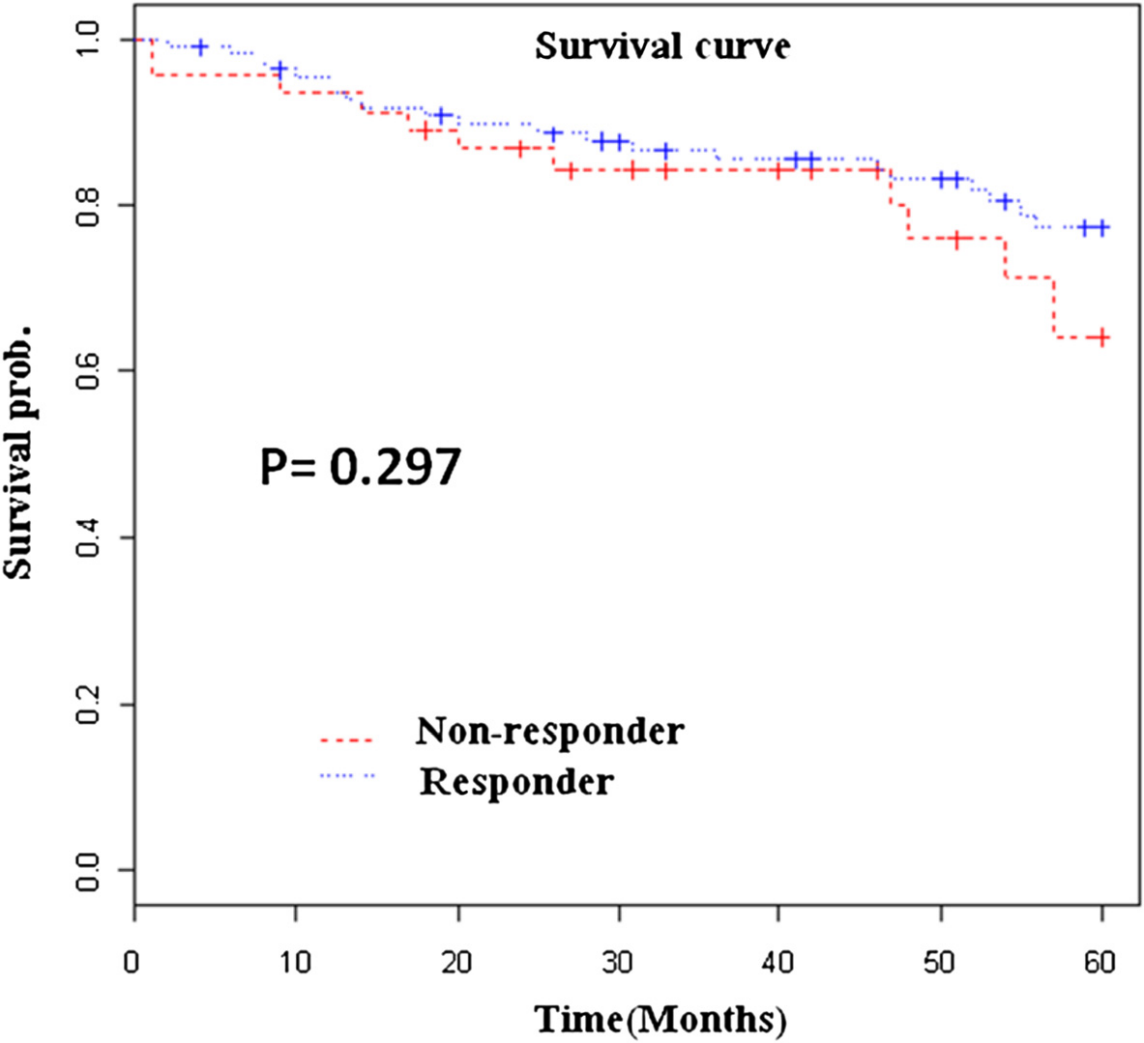
Cumulative survival of HBV vaccine responders and non-responders over 5 years.

We followed up on all patients after the vaccination to investigate the association between seroconversion and mortality. Figure 1 shows that there was no significant survival difference for responders and non-responders (P = 0.297). A Cox regression analysis, which considered patient age, presence of DM, initial serum albumin levels, seroconversion after HBV vaccination, dialysis modality, and gender, showed that advanced age and malnutrition were associated with increased mortality (P = 0.035 and P = 0.032, respectively) (Table 2).

**Table 2:** Cox proportional hazards regression of immune response to HBV vaccination and mortality

**Parameter**	**P- value**
Age	0.035
Modality	0.210
Seroconversion of HBV vaccination	0.600
Albumin	0.032
Gender	0.060
Diabetes Mellitus	0.670

We also evaluated the association between one-month post-vaccination anti-HBs titers and mortality. There was no significant survival difference between immune sustainers and non-sustainers (P = 0.201). Cox regression analysis indicated that only age and low levels of albumin were associated with mortality (p = 0.04 and p = 0.032, respectively).

**Figure 2:**
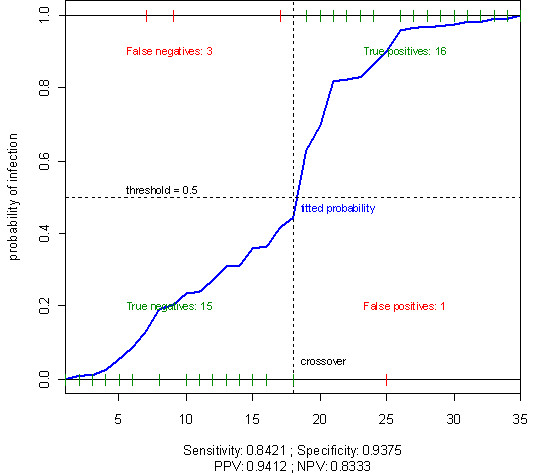
The ROC curve shown used to examine the performance of logistic regression model.

The predictive model of mortality due to infection in dialysis patients demonstrated that non-response, DM, age, and low levels of albumin all predicted significantly higher mortality rates due to infection (Table 3). This model performs well and has high sensitivity and high specificity (sensitivity = 0.842, specificity = 0.938, positive predictive values = 0.941, negative predictive value = 0.833). The area under the ROC curve (AUC) provides an overall measure of the model’s classification accuracy, with a value of one representing perfect accuracy. The ROC curve shows a high capacity for discriminating infection-cause mortality vs. non-infection cause mortality, with an AUC = 0.9112 (Figure 2). The immune response following HBV vaccination and other co-factors were not associated with CV mortality (data not shown).

**Table 3:** The predictive model of infection-cause mortality in dialysis patients Logit (probability of infection-cause mortality) =β0 + β1*Retiter + β2*DM + β3*Age + β4*Albumin + β5*DM*Albumin + β6*Age*Albumin

**Covariates**	**Coefficient**	**p-value**
Intercept	143.4122	0.0232
Seroconversion following HBV vaccination	−4.7392	0.0068
Presence of DM	−5.3569	0.0084
Age	−1.9758	0.3333
Albumin	−40.2689	0.0234
DM*Albumin	13.1720	0.0089
Age*Albumin	0.5699	0.0317

## Discussion

In this study, we showed that the seroconversion rate of our dialysis patients was 70.5% after four double doses (40 μg per dose) of the hepatitis B vaccine administered over 6 months. We also found that responders were significantly younger than non-responders (P = 0.003). Seroconversion to HBV vaccination and one-month post-vaccination anti-HBs titers did not predict all-cause mortality in our maintenance dialysis patients. After adjusting for age, DM, albumin levels, gender, and dialysis modality, non-seroconversion following HBV vaccination could independently predict mortality due to infection in ESRD dialysis patients. This novel and interesting finding demonstrates the possible relationship between the vaccination response and the specific cause of mortality in ESRD patients.

Our study dialysis subjects displayed a similar suboptimal immune response to patients in previous studies. In ESRD patients, the response rate to HBV vaccination is approximately 50%–80%, which is lower than the 90% response rate in the general population, with advanced age being a significant negative factor associated with this poor immune response [11-13]. Numerous factors associated with the impaired immune response following HBV vaccination in ESRD, such as age, albumin levels, HCV infection, and gender, have been well studied [8-10,13]. Current studies have focused on the insufficient acquired immunity due to uremia to account for the suboptimal response rates following HBV vaccination [14]. Recent studies have proposed that the immunodeficiency in ESRD results from the impaired function of antigen-presenting cells, alteration of toll-like receptors, weakened activation of T lymphocytes, and the imbalance of T-helper lymphocytes (Th 1/Th 2 ratio) [14-16].

In the present study, we demonstrated that DM, age, low albumin levels, and non-response following HBV vaccination could predict mortality due to infection.

Several negative determinants of mortality in ESRD, such as old age, DM, and malnutrition, have been documented [7]. Immune dysfunction in uremia is also recognized as a determinant of mortality. However, our findings are important because they demonstrate a link between infection-cause mortality and insufficient immune responses to HBV vaccination in ESRD, since these two clinical presentations involve homogenous but varied interactions between the immune, innate immune and the adaptive immune systems. A recent review indicated that in dialysis patients innate immunity plays an important role in fighting infections, while acquired immunity (via antibody production) also makes a significant contribution [14]. We therefore hypothesize that T cell dysfunction may be the main link between the high mortality due to infection and the insufficient antibody response to vaccination in ESRD patients. Impaired cytotoxic CD8 T cell response limits the fight of the infected host defense system against the pathogens, while the inadequate function of helper CD4 T cells weakens the humoral immune system. The results of studies by Alvarez-lara et al. and Meier P et al. support this hypothesis, finding increased apoptosis of T cells in uremia [17-19]. An alternative interpretation is that the key linking factor between high mortality due to infection and the impaired vaccination response lies upstream of T-cell activation, i.e., the pattern of recognition and the antigen presenting cell or secondary dysregulation or activation of cytokines [6,14,20].

It has been proposed that both the innate and acquired immune systems may play a role in the progression of atherosclerosis, the major pathogenic factor responsible for most deaths in ESRD patients [21]. Interestingly, we did not find an association between seroconversion and all-cause mortality or between seroconversion and CV-cause mortality in dialysis patients. The correlations among persistent inflammation, CVD, immune dysfunctions, and infections in ESRD have become apparent in recent years [5,6,22-24].

Chronic inflammation as a result of hypercytokinemia and an imbalance in the pro-inflammatory to anti-inflammatory ratio has been well recognized in ESRD [25]. The dysregulation of various cytokines due to the uremia milieu may link inflammation-atherosclerosis to impaired immunity inflammation in dialysis patients [14]. Several studies have attempted to link the levels and types of cytokines with clinical outcomes. Girndt et al. determined that higher levels of IL-6 and TNF-α correlated with an immunocompromised status, that is, non-seroconversion to HBV vaccinations in HD patients [26]. Kimmel et al. assessed immune parameters such as cytokines in 230 HD patients, with a three-year follow up. Their results showed that increased levels of IL-6, TNF-α, IL-1, and IL-13 correlated with a higher relative mortality risk. High levels of IL-2, IL-4, IL-5, and IL-12, and T cell functions were associated with survival in HD patients. The authors did not analyze the causes of mortality in their patients or further investigate the associations between the causes of mortality and cytokines. Interestingly, they noted that the patterns of cytokines involved may be more important than the individual cytokine levels in interpreting survival outcomes [27].

Among the other studies on cytokines’ ability to predict mortality in ESRD, the study by Badiou et al. measured levels of both pro-inflammatory cytokines and anti-inflammatory cytokines in 134 HD patients and found that IL-6 levels could strongly predict CV mortality. In addition, (IL-4+ IL-6+ IL-10)/(IL-2 + IFN-γ) was associated with non-CV mortality. The authors speculated that examination of the Th1/Th2 cytokine relation might be more relevant for predicting non-CV mortality [28]. These findings indicate that the laboratory immune parameter, especially the Th1/Th2 cytokine ratio that implies defective immunity, may be able to predict clinical outcomes. The clinical immune parameters and the immune response following HBV vaccination may be able to correlate with mortality in ESRD patients.

The study by Fernandez et al. of 64 HD patients noted that low levels of albumin negatively influenced the response to HBV vaccination. In the study’s survival analysis of 31 patients, non-responders had higher mortality and morbidity [29]. The authors did not adjust for albumin levels or age in the survival analysis of these HD subjects. However, our Cox regression survival analysis of 156 dialysis patients reveals that only old age and low levels of albumin have significantly higher mortality but that non-response following HBV vaccination did not. Since low albumin levels are recognized as an independent factor of mortality in ESRD [30], we speculate that low albumin levels account for the concomitant impaired immune response following HBV vaccination and increased mortality in the Fernandez et al. study.

The Kimmel et al. study suggests that better T cell function and humoral immunity are associated with a survival advantage in HD patients [27]. Unlike the predictive value of the cytokine ratio on mortality mentioned above, our study demonstrated no significant relationship between the clinical immune response following HBV vaccination and all-cause mortality. The levels and types of cytokines in dialysis patients were the result of the interacting influences of residual renal function, dialysis adequacy, comorbidity, and current treatment [31]. Our dialysis patients received HBV vaccinations at the initiation of renal replacement therapy. Some of our study subjects, especially the PD patients, retained some residual renal function, which would affect the immune response to HBV vaccinations. In the Badiou et al. and Kimmel et al. studies, the levels of circulating cytokines (either Th1 or Th2-related) were significantly higher in the HD patients than in the control patients [27,28]. The effects of these unusual levels of circulating cytokines may not correlate well with their baseline local immunological functions [31]. These circulating cytokines may operate in a more sophisticated pathophysiologic network, causing erythropoietin resistance, hypoalbuminemia, frailty, atherosclerosis, and dyslipidemia [31,32]. Therefore, the clinical outcomes, survival and immune response following HBV vaccination may be the diverse results of the differing interactions of various cytokines at specific cellular, organ, and system targets. We therefore could not determine whether a direct association exists between seroconversion and all-cause mortality in dialysis patients despite the well-recognized immunodeficiency, hypercytokinemia, and high mortality in ESRD populations [1,14,24,33,34].

We did not check the antibody of the core HBV antigen (anti-HBc). The finding of a positive anti-HBc in the absence of HBs Ag or anti-HBs is relatively uncommon [35]. Chen et al. found that the responsiveness rates of the hepatitis B vaccination were the same between isolated anti-HBc positive and normal subjects [36].

We recognize that our study has certain limitations, such as the relatively small number of dialysis patients included to determine the infection-based and CV mortality. Moreover, infection was the leading cause of mortality among our study subjects, despite being the second leading cause of deaths in the ESRD population as a whole. The immune status of dialysis patients may change after losing residual renal function. The results of our study need to be replicated in prospective studies of using larger populations of chronic, stable dialysis patients.

## Conclusions

In conclusion, we demonstrated that non-response following HBV vaccination might be associated with infection-cause mortality in our dialysis patients. However, the clinical immune response following HBV vaccination would not be associated with all-cause mortality in our dialysis patients.

## Competing interests

The authors declare that they have no competing interests.

## Authors’ contributions

SL carried out the data collection and drafted the manuscript. JL, SW, and I W participated in the design of the study. CT, HL, CC, and CH performed the statistical analysis. YL conceived of the study, and participated in its design and coordination. All authors read and approved the final manuscript.

## Pre-publication history

The pre-publication history for this paper can be accessed here:

http://www.biomedcentral.com/1471-2369/13/97/prepub
